# An Analysis of the Cost Variation Among Different Antimicrobial Agents: The Indian Scenario

**DOI:** 10.7759/cureus.64538

**Published:** 2024-07-14

**Authors:** Akash Shah, Chetna Patel, Paras Shah, Jaykumar Patel, Brijesh Sojitra, Sajal Pandya, Aarmin Shaikh

**Affiliations:** 1 Pharmacology, Government Medical College, Surat, Surat, IND; 2 Pharmacology, Government Medical College and New Civil Hospital, Surat, Surat, IND; 3 Pharmacology and Therapeutics, Government Medical College, Surat, Surat, IND

**Keywords:** cims, % cost variation, cost ratio, antimicrobial agents, cost variation analysis

## Abstract

Background and objective

Infectious diseases pose a substantial global health challenge, especially in developing countries where healthcare accessibility is limited. Pharmaceutical expenses constitute a significant share of out-of-pocket expenditure (60-90%). Hence, the affordability of medications becomes a critical determinant for patient compliance. This study focuses on the economic dynamics of antimicrobial agents.

Methodology

After collecting data from the Current Index of Medical Specialties (CIMS), different antimicrobial agents (AMAs) were assessed based on their cost per 10 tablets/10 capsules/one vial of injection. A comprehensive analysis was performed to assess the minimum and maximum costs for each medication across diverse pharmaceutical companies. Cost variation was assessed through both the cost ratio and percentage cost variation. The data were analyzed and represented using descriptive statistics

Results

Our findings indicate significant cost variations, with nitrofurantoin 100 mg tablet showcasing a staggering 1498.5% variation, followed by meropenem 500 mg vial at 473.91%. Conversely, the cotrimoxazole (sulfamethoxazole 800 mg + trimethoprim 160 mg) tablet exhibits a minimal 6.05% variation, underscoring the diversity in pricing strategies. The number of brands ranged from two to 62.

Conclusions

This study underscores the importance of considering cost variations in antimicrobial agents while prescribing the same. Doing so will not only address the economic challenges faced by patients but also help in improving compliance and reducing the risk of antimicrobial drug resistance. This approach advocates for a more economically sustainable and patient-centric healthcare ecosystem in India.

## Introduction

There are currently over 3,000 pharmaceutical companies, more than 10,000 manufacturing units, and more than 60,000 generic brands across 60 therapeutic categories in the Indian pharmaceutical market [1,2]. This situation has led to increased price variation among marketed drugs [3]. Pharmacoeconomics plays an important role in the practice of medicine [4]. ‘Cost analysis’ is a type of partial pharmacoeconomic evaluation where the costs of two or more drugs are compared without considering the outcome [5]. Pharmaceutical costs are rising faster than any other healthcare expense. The price of treatment is important in health economics, especially in developing countries like India, where limited financial resources affect all areas of medicine. Price discrimination in the pharmaceutical industry is a global issue, including in India. Despite having some of the cheapest drug prices in the world, approximately 65% of the Indian population lacks access to essential medicines due to factors such as inadequate healthcare infrastructure and supply chain issues [6].

There are many challenges in treating a range of infectious diseases like tuberculosis, malaria, respiratory infections, and dengue fever in India, which can cause significant mortality and morbidity [7]. The availability of life-saving antimicrobials like ciprofloxacin, vancomycin, and meropenem is crucial for disease cure, and many infectious diseases are curable if treated rationally. Antibiotics are commonly prescribed in almost all hospital departments. The WHO Guide to Good Prescribing emphasizes the "P" for personal, indicating that physicians should have a personal formulary of drugs prescribed rationally, considering efficacy, safety, suitability, and cost. Physicians must also make minor adjustments to the standard dose, frequency, or route of drug administration and assess the cost-effectiveness of the prescribed drug [8].

However, antimicrobial agents (AMAs) are costlier preparations and the absence of comparative price information makes it difficult for physicians to prescribe the most economical treatment regimen, leading to the unnecessary prescription of more expensive drugs [9]. While this may not be a concern in developed countries with medical insurance systems, the affordability of AMAs is a major concern in developing countries like India, where most of the population is uninsured and 50-90% of healthcare expenses are paid out-of-pocket [7,10]. Prescribing costly brands of AMAs increases treatment costs, potentially making it difficult for patients to afford a complete course, thus contributing to poor compliance and the development of antimicrobial resistance. A survey revealed that a notable proportion of high-income participants also reported non-compliance due to costs. The link between poor antimicrobial compliance and antimicrobial resistance is well-documented, particularly in chronic infections like tuberculosis [11].

Misuse and overuse of AMAs are also common in India, where most drugs are available in branded forms. The pharmaceutical industry manufactures many branded formulations of the same drug with significant price differences. Little attention is paid to cost variations among different brands of reputable pharmaceuticals. There is an urgent need to reduce price discrepancies among drug formulations in the Indian market as well as globally. Reducing prescription costs can significantly improve overall healthcare outcomes. Understanding the variability in pricing among different generics from various companies is imperative to select the most cost-effective therapy, which can significantly impact long-term compliance and overall healthcare outcomes [12].

To address the issues related to the affordability and availability of medicine, the Government of India introduced the Drug Price Control Order (DPCO) in 2013. This order aimed to make essential drugs more affordable. As part of the order, the National Pharmaceutical Pricing Authority revised and set the prices for 814 essential medicines [1]. This study aims to analyze the cost differences between different brands of the same generic antimicrobial agent. The objective is to recommend a cheaper, more effective brand to improve patient compliance and reduce drug and healthcare costs.

## Materials and methods

This observational study involved obtaining information on the cost of different brands of AMAs available in India from the latest edition (Jan-Apr 2024) of the Current Index for Medical Specialties (CIMS).

Inclusion and exclusion criteria

AMAs commonly used in our hospital were included in the study. Drug formulations manufactured by only one company, drug formulations lacking price information, and drugs other than antimicrobial agents were excluded from the study. Different doses of the same antimicrobial agents were also excluded.

Cost evaluation and variation analysis

A comprehensive analysis of a specific AMA was conducted by documenting the minimum and maximum costs in Indian rupees (INR) across various pharmaceutical companies producing the same strength of the medication. The cost evaluation was based on the price of 10 tablets, 10 capsules, or one injection. Cost variation was analyzed by calculating the cost ratio and the percentage cost variation. Additionally, we assessed costs per defined daily dose (DDD), detailing the minimum and maximum costs per DDD for each antimicrobial agent included in the study.

1) $Cost ratio = Maximum Cost\div Minimum Cost$

2) $Percentage Cost variation = [(Maximum Cost-Minimum Cost) \times 100] \div Minimum Cost$

Statistical analysis

A descriptive statistical analysis was performed. The findings were expressed as absolute numbers and percentages.

## Results

A total of 16 antimicrobial agents commonly used for treating bacterial infections were included in the analysis. The study showed a large difference in the costs of various brands of the same antimicrobial agents (Table 1). The number of brands showed an average (mean) of 20.93 with a substantial standard deviation of 20.42, indicating significant variability, and a median of 11, suggesting a skewed distribution with a range from two to 62. The cost ratio had a mean of 3.89 and a standard deviation of 3.55, with a median of 3.16, implying a moderately spread distribution, ranging from 1.06 to 15.99. The percentage cost variation has a high mean of 288.8 and a large standard deviation of 355.23, highlighting considerable variability, with a median of 215.84 and an extensive range from 6.05 to 1498.5. The data suggests considerable variability in the number of brands and cost ratios, along with notable fluctuations in the percentages of cost variation.

**Table 1:** Analysis of the cost variation among various antimicrobial agents SD: standard deviation

	Mean ± SD	Median	Range
Number of brands	20.93 ± 20.42	11	2-62
Cost ratio	3.89 ± 3.55	3.16	1.06-15.99
Percentage cost variation	288.8 ± 355.23	215.84	6.05-1498.5

The number of brands ranged from two for cotrimoxazole (sulfamethoxazole 800 mg + trimethoprim 160 mg) tablets and gentamicin 20 mg vials to 62 for amoxicillin 500 mg + clavulanic acid 125 mg tablets and cefixime 200 mg tablets. When comparing the costs of different brands of individual antimicrobial drugs, we found significant variations, e.g., the highest cost ratio was 15.99 for nitrofurantoin 100 mg tablets, followed by 5.74 for meropenem 500 mg vials and 5.62 for cefosulbactam (cefoperazone 1000 mg + sulbactam 500 mg) vials. In contrast, the smallest cost ratio was 1.06 for cotrimoxazole (sulfamethoxazole 800 mg + trimethoprim 160 mg) tablets, followed by 1.10 for clindamycin 300 mg capsules and 1.39 for vancomycin 500 mg vials. The price range between the cheapest and most expensive brands of individual antimicrobials varied widely, such as from 329 to 1888.15 for meropenem 500 mg vials, from 223.36 to 799 for piperacillin 4000 mg + tazobactam 500 mg vials, and from 110 to 617.92 for cefoperazone 1000 mg + sulbactam 500 mg. The minimum price range was found to be 16.03-17 for cotrimoxazole (sulfamethoxazole 800 mg + trimethoprim 160 mg) tablet followed by 3.59-6.58 for gentamicin 20 mg vial and 220-242.65 for clindamycin 300 mg capsule (Table 2).

**Table 2:** Cost analysis of different antimicrobial agents ^*^Maximum value. ^^^Minimum value INR: Indian rupee

Category	Drug	Defined daily dose	Number of brands available	Min cost (INR)	Max cost (INR)	Cost ratio	Cost variation (%)
Oral (combination)	Amoxicillin+ clavulanic acid	500 mg/125 mg	62^*^	101.68	380	3.74	273.72
Oral (single)	Cefixime	200 mg	62^*^	53.1	200	3.77	276.75
Oral (single)	Linezolid	600 mg	10	201	499	2.48	148.26
Oral (single)	Ciprofloxacin	500 mg	20	16.87	67	3.97	297.15
Oral (single)	Azithromycin	500 mg	9	116.7	280	2.40	139.93
Oral (single)	Nitrofurantoin	100 mg	9	10	159.85	15.99^*^	1498.5^*^
Oral (combination)	Cotrimoxazole	800 mg/160 mg	2^^^	16.03	17	1.06^^^	6.05^^^
Parenteral (single)	Meropenem	500 mg	12	329	1888.15	5.74	473.91
Oral (single)	Doxycycline	100 mg	5	8.46	41.02	4.85	384.87
Oral (single)	Clindamycin	300 mg	5	220	242.65	1.10	10.30
Parenteral (combination)	Piperacillin + tazobactam	4 g/500 mg	40	223.36	799	3.58	257.72
Parenteral (single)	Ceftriaxone	1 gm	43	50	98	1.96	96
Parenteral (single)	Amikacin	100 mg	20	13.25	36.3	2.74	173.96
Parenteral (single)	Vancomycin	500 mg	5	267	370.19	1.39	38.65
Parenteral (single)	Gentamicin	20 mg	2^^^	3.59	6.58	1.83	83.29
Parenteral (combination)	Cefoperazone + sulbactam	1 g/500 mg	29	110	617.92	5.62	461.75

**Figure 1:**
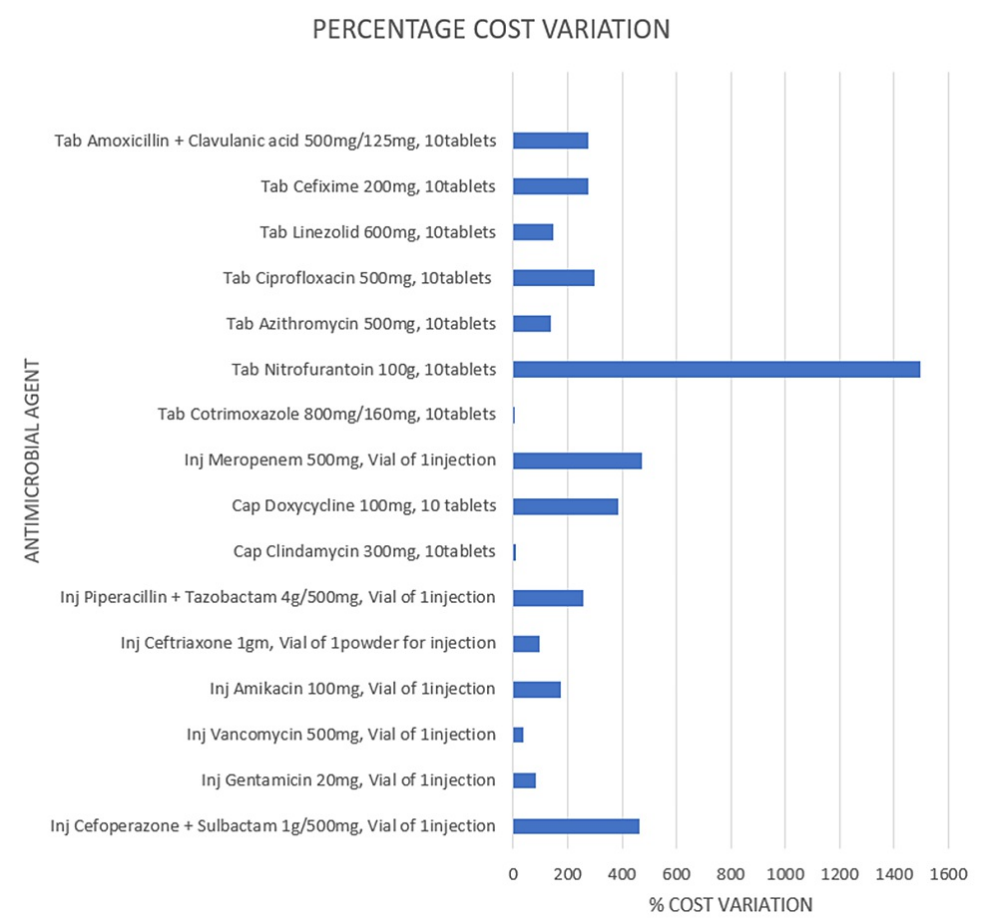
Percentage cost variation of different antimicrobial agents

The percentage cost variation was calculated as per the formula mentioned earlier. The percentage cost variation was highest at 1498.5% for nitrofurantoin 100 mg tablets, followed by 473.91% for meropenem 500 mg vials and 461.75% for cefosulbactam (cefoperazone 1000 mg + sulbactam 500 mg) vials. The lowest percentage cost variation was for cotrimoxazole (sulfamethoxazole 800 mg + trimethoprim 160 mg) tablets at 6.05%, followed by 10.30% for clindamycin 300 mg capsules and 38.65% for vancomycin 500 mg vials (Figure 1).

## Discussion

The rising cost of medical care and limited resources have led to a surge in pharmacoeconomic studies in India. Key factors for the rise in pharmacoeconomic studies in India include aging, chronic diseases, new drugs, new uses for existing drugs, and the growth of the pharmaceutical industry. These studies aim to demonstrate pharmacoeconomic value by balancing economic, humanistic, and clinical outcomes [13]. In 2000, India implemented the "Health for All" policy, which aimed to make quality medicines accessible at affordable prices [14]. Previous studies on medications such as antidiabetics, antitubercular drugs, and antihypertensives have highlighted significant price variations among different brands with the same active ingredients [15-17]. While the effectiveness and safety of drugs are well-known, their pricing is often ignored and undervalued [18]. The availability and affordability of drugs influence prescription decisions [19]. The high cost of AMAs can be a significant barrier to patient compliance, especially in low-income populations. When patients cannot afford their medications, they may take them irregularly or stop altogether, thereby increasing the risk of relapse and contributing to the spread of antimicrobial resistance.

In India, numerous brands and formulations of AMAs are available, with a substantial variation in their prices. Pharmaceutical companies have developed new strategies to circumvent DPCO. They modify the formulation compositions by altering excipients or creating dosage forms that are not listed on the scheduled drug list. Consequently, only 14-17% of the Indian pharmaceutical market is under price control, resulting in significant brand price variation in the non-controlled segment. Scheduled drugs, with prices capped by the government, allow a 16% margin for retailers and an 8-10% margin for distributors. In contrast, for non-price-controlled drugs, pharmaceutical companies have the freedom to set margins, often leading to excessively high retail prices [20].

People often fail to notice the big price differences between affordable and expensive medications. Issues like lack of information among doctors, government rules including drug pricing policies, and the economic interests of drug companies lead to the prescribing of more expensive drugs when less costly drugs are available [9]. This often makes patients stop following their treatment. To enhance adherence, clinicians should select the least expensive medication. Since no scientific evidence has established the superiority of the most expensive brand over the cheapest one, conscientious prescribers should always opt for the least costly brand when prescribing.

We assessed 12 single-drug antibiotics and four fixed-dose combinations (FDCs) and found large cost differences between different brands of essential medicines. In our study, the smallest cost variation was 6.05%, and the largest was 1498.5%. The wide range of cost variation from 6.05% to 1498.5% can be attributed to several factors, including differences in manufacturing and production costs, marketing and distribution expenses, and regulatory and approval costs. Also, brand perception, economic scale, and competition play significant roles. Established brands may command higher prices due to perceived quality and brand loyalty, while larger manufacturers can offer lower prices through economies of scale. The smallest variation is similar to a previous study in India by Meena et al., where the smallest cost variation was 7.34% for ciprofloxacin. The largest variation in their study was 1049.82% for azithromycin, which is lower than what we found [1]. Another study by Mir et al. reported the largest cost variation of 2000% for ciprofloxacin, which is higher than in our study [11]. In our study, the highest cost ratio was 15.99 for nitrofurantoin 100 mg tablets, which differs from previous reports where the highest cost ratios were 11.4 and 19.3, respectively [1,11].

Quality data on individual drug brands is not easily accessible to the public, making it challenging to obtain this information. When formulating policies, the government should thoroughly examine these price variations among antibiotic brands and implement measures for price regulation. Prescribers should take drug costs into account in their practice to alleviate the financial burden on patients, while simultaneously focusing on other factors like efficacy and safety. The generic drug scheme of the government, Pradhan Mantri Bharatiya Janaushadhi Pariyojana (PMBJP), with more than 10,000 Janaushadhi Kendras across the country, was launched to make low-priced, good-quality medicines available for all. All generic drugs under the scheme are procured and supplied by the Pharmaceuticals & Medical Devices Bureau of India (PMBI) to ensure quality and efficacy. However, the same cannot be ensured for cheap brands available in the market [21].

The Government of India has insisted that doctors prescribe drugs by their generic name. However, that does not ensure that brands with lower costs will be dispensed by the pharmacist [22]. There is a widespread belief among people and dispensing chemists that a branded product is better in terms of quality and safety than the generic variant [23]. Pharmaceutical companies tend to promote the misconception that higher-priced products are superior to their cheaper counterparts, which is not true. Therefore, an effective system to regulate price variations among brands should be implemented to reduce and control the cost of branded drugs. This system should also consider both direct costs, such as the price of the drugs themselves, and indirect costs, including hospitalization, additional treatments due to side effects, and loss of productivity.

Limitations

This study has certain limitations. We did not analyze pediatric and topical antimicrobial preparations. Another limitation is that we compared drug costs without considering their quality. Also, the study focused on direct cost variations among different brands of antimicrobial agents without taking indirect costs into account.

## Conclusions

This study reveals a substantial cost variation among different brands of nitrofurantoin, meropenem, and cefoperazone + sulbactam. Taking the cost of AMAs into account while prescribing them can alleviate the economic burden on patients, enhance patient compliance, and mitigate the risk of antimicrobial drug resistance development. Further research is crucial to comprehensively understand how variations in drug costs influence clinical outcomes, particularly in terms of treatment efficacy and patient outcomes. Exploring the relationship between cost variations and clinical outcomes through rigorous research will provide valuable insights for healthcare providers and policymakers, guiding strategies to optimize treatment outcomes while managing healthcare expenditures effectively.

Policy initiatives like implementing price caps, transparent pricing mechanisms, subsidies for patients requiring costly antimicrobial treatments, educational campaigns, encouraging evidence-based prescribing, and promotion of generic drug use can not only reduce the economic burden on healthcare systems and patients but also foster more sustainable antimicrobial stewardship practices.
